# Intrinsic Levanase Activity of *Bacillus subtilis* 168 Levansucrase (SacB)

**DOI:** 10.1371/journal.pone.0143394

**Published:** 2015-11-23

**Authors:** Luz Méndez-Lorenzo, Jaime R. Porras-Domínguez, Enrique Raga-Carbajal, Clarita Olvera, Maria Elena Rodríguez-Alegría, Ernesto Carrillo-Nava, Miguel Costas, Agustín López Munguía

**Affiliations:** 1 Departamento de Ingeniería Celular y Biocatálisis, Instituto de Biotecnología, Universidad Nacional Autónoma de México, Cuernavaca, Morelos, México; 2 Laboratorio de Biofisicoquímica, Departamento de Fisicoquímica, Facultad de Química, Universidad Nacional Autónoma de México, Distrito Federal, México; Oak Ridge National Laboratory, UNITED STATES

## Abstract

Levansucrase catalyzes the synthesis of fructose polymers through the transfer of fructosyl units from sucrose to a growing fructan chain. Levanase activity of *Bacillus subtilis* levansucrase has been described since the very first publications dealing with the mechanism of levan synthesis. However, there is a lack of qualitative and quantitative evidence regarding the importance of the intrinsic levan hydrolysis of *B*. *subtilis* levansucrase and its role in the levan synthesis process. Particularly, little attention has been paid to the long-term hydrolysis products, including its participation in the final levan molecules distribution. Here, we explored the hydrolytic and transferase activity of the *B*. *subtilis* levansucrase (SacB) when levans produced by the same enzyme are used as substrate. We found that levan is hydrolyzed through a first order exo-type mechanism, which is limited to a conversion extent of around 30% when all polymer molecules reach a structure no longer suitable to SacB hydrolysis. To characterize the reaction, Isothermal Titration Calorimetry (ITC) was employed and the evolution of the hydrolysis products profile followed by HPLC, GPC and HPAEC-PAD. The ITC measurements revealed a second step, taking place at the end of the reaction, most probably resulting from disproportionation of accumulated fructo-oligosaccharides. As levanase, levansucrase may use levan as substrate and, through a fructosyl-enzyme complex, behave as a hydrolytic enzyme or as a transferase, as demonstrated when glucose and fructose are added as acceptors. These reactions result in a wide variety of oligosaccharides that are also suitable acceptors for fructo-oligosaccharide synthesis. Moreover, we demonstrate that SacB in the presence of levan and glucose, through blastose and sucrose synthesis, results in the same fructooligosaccharides profile as that observed in sucrose reactions. We conclude that SacB has an intrinsic levanase activity that contributes to the final levan profile in reactions with sucrose as substrate.

## Introduction

Levan is a fructose polymer composed by β2–6 linkages in the main chain with branching in β2–1 bonds [1]. It is synthesized by levansucrases (EC 2.4.1.10) (LS) which belong to family 68 of glycoside hydrolases. LSs have a five-bladed β-propeller fold in a single-domain structure enclosing a funnel-like central cavity where the active site is located [2].

It is known that in the presence of sucrose, *B*. *subtilis* levansucrase (SacB) forms a covalently bound fructosyl-enzyme complex which later gives rise to two type of reactions: *transfructosylation*, when the fructosyl residue is transferred to sucrose, to the growing levan chain or to an acceptor molecule added to the reaction medium [3–5] and *hydrolysis*, when the fructosyl residue is transferred to water. The levanase activity of *B*. *subtilis* levansucrase was described as part of the enzyme properties [4, 6–8]. However, little efforts have been made to further explore this activity and its role in the overall levansucrase reaction specificity. Indeed, the initial reports dealing with levansucrase properties defined reaction conditions in which levansucrase hydrolyzes levans. Specifically, it was reported that levanase activity of levansucrase occurs particularly with low molecular weight levans and is limited by the β2–6 linkages found in the branching points of the polymer chain which the enzyme was unable to hydrolyze [6, 7]. According to these authors, levans of molecular weights higher than 10^6^ Da are not substrates for levansucrase hydrolysis. The authors also demonstrated the *disproportionation* activity of levansucrase, incubating the enzyme with levano-pentaose and observing the production of higher and lower degrees of polymerization (DP) fructooligosaccharides. Levanase activity is not exclusive of levansucrase from *B*. *subtilis*, as this activity has also been observed in levansucrase from *Lactobacillus reuteri* [9], *Zymomonas mobilis* and *Rahnella aquatilis* [10]. Levanase activity of levansucrase may become important at the end of the reaction when, in the absence of sucrose, levan molecules may be used as substrate [9].

As far as the kinetic properties of the levanase activity of levansucrase is concerned, Rapoport and Dedonder (1963) report a *K*_*m*_ value of 5 x 10^−3^ M for low molecular weight levan, which they found similar to the affinity value for levans when used as initiators in reactions in the presence of sucrose. Chambert *et al*. (1974) proposed a kinetic model derived from the Ping-Pong mechanism of fructose transfer, which included an exo-type mechanism for the hydrolysis of levan and reported a first order rate constant of 50 s^-1^ for the hydrolysis of levan molecules of any size. In the same model, a *k*_*cat*_ of 1.4 x 10^3^ s^-1^ was reported for levan synthesis, which they found 30 times faster than the hydrolysis at 22°C. A decade later, Yamamoto *et al*. (1985) found that Michaelis-Menten type kinetics describe the hydrolytic reaction with *k*_*cat*_ values of 3.72 x 10^−3^ and 5.7 x 10^−3^ s^-1^ for the hydrolysis of high and low molecular weight levan, respectively. According to Dedonder [7], levansucrase cannot hydrolyze the smallest fructans 6-kestose (β-D-fructofuranosyl-(2→6)-β-D-fructofuranosyl-(2→1)-α-D-glucopyranoside and the disaccharide levanobiose (β-D-fructofuranosyl-(2→6)- β-D-fructofuranoside); levansucrase can neither hydrolyse β2–1 linkages present in inulin-type fructans.

It is known that, depending on reaction conditions, levan produced by *B*. *subtilis* 168 levansucrase is synthesized as a wide bi-modal distribution of polymer chains, corresponding to high (3,500 kDa) and low (8.3 kDa) average molecular weight products, hereafter referred to as high molecular weight (HMw) levan and low molecular (LMw) weight levan, respectively [1, 11]. In spite of the early research carried out regarding the hydrolytic activity, subsequent research on this aspect of levansucrase specificity has been scarce, particularly concerning its effect on the stability and profile of the synthesized product, as well as its eventual role in the final levan molecules distribution. The aim of this study was to explore various features related with the activity of the *B*. *subtilis* levansucrase when levan is used as substrate.

## Materials and Methods

### Sac B production and purification

Levansucrase (SacB) from *Bacillus subtilis* 168 (ATCC^®^ 23857^™^) was expressed in *Escherichia coli* BL21 and incubated in Luria-Bertani medium with 0.2 mg/mL of ampicillin at 37°C and 200 rpm until an OD_600_ of 0.5. The culture was induced with 0.2 mM IPTG (Research Organics) for 8 h at 18°C and 120 rpm. Cells were harvested by centrifugation and lysed with a solution of lysozyme 1 mg/mL (Sigma), sonicated, and centrifuged. The enzyme was purified using ion exchange chromatography with an AKTA prime (Amersham Pharmacia Biotech) system in 1 M phosphate buffer gradient at a flow of 1 mL/min for 20 min. Finally, the eluted enzyme was dialyzed against 50 mM sodium acetate buffer, 1 mM CaCl_2_ and pH 6.0 (working buffer). Exoinulinase purified from *Bifidobacterium longum* extracts was applied for a single experiment to demonstrate the presence of β2–1 linkages.

### Kinetic parameters (initial rates)

Levan hydrolysis activity was determined by measurement of fructose released from HMw levan and LMw levan. The polymers were produced following the method described by Porras-Dominguez et al [11]. Initial reaction rates at 37°C were measured by following the reducing power released from the working mixture containing a SacB concentration of 0.126 mg/mL (2.4 μM) and LMw levan ranging from 0.83 to 249 mg/mL in the working buffer. In the case of HMw levan, the concentrations range was limited to a maximum of 40 mg/mL due to levan solubility and medium viscosity. Both concentrations of levans are also reported based on total fructose equivalents in order to measure reaction conversion: X = (fructose released/total fructose in the substrate). The initial rate of levan hydrolysis is reported in μmol of fructose residues released per minute per mL. When molar concentration of levans were required, the average molecular weight of the levan distribution was considered: HMw levan = 3,500 kDa and LMw levan = 8.3 KDa. The data were fitted to the Michaelis Menten equation using non-linear regression with the software Sigma Plot Version 12.0 (Systat Software, San Jose, CA).

Acceptor reactions with levan as fructosyl donor were carried out under the above mentioned conditions with 2.5mM of levan in the presence of 148 mM of fructose or 127mM glucose. These conditions approach those found towards the end of a 100g/L of sucrose, based on 60% of sucrose hydrolysis (160mM of fructose) and 2.5mM of 8.3 kDa levan (40% transferase).

### Enzyme kinetics determined by isothermal titration calorimetry

Levanase activity of levansucrase was also studied in an Isothermal Titration Calorimeter (ITC) VP-ITC Micro Cal (Northampton, MA, USA) with the single injection method, in which the rate of reaction is followed after a large addition of substrate (placed in the syringe) into the cell of the calorimeter where the enzyme is previously placed [12,13]. Prior to the injection of the substrate, all solutions were degassed with stirring under vacuum and were allowed to reach thermal equilibrium. All measurements were carried out at 25°C. In the ITC experiments, a 40 μL single injection of 30.16 mM LMw levan is made into the cell containing SacB at different known concentrations (in the range 0.5 to 5 μM). Hence, in all the titrations carried out the concentration of substrate was much higher than the concentration of the protein in the cell. In order to correct the data for the heat of dilution of the substrate in the syringe, in a separate experiment the heat resulting from a single levan solution injection into the working buffer was measured and later subtracted from the heat of reaction.

In the ITC calorimeter, the thermal power is measured as a function of time. This thermal power *dQ/dt* (or heat rate) is directly proportional to the instantaneous reaction rate (*v*) according to:

$$v=\frac{1}{V_{cell}{\Delta H}_{r}}\;\frac{dQ}{dt}$$

where *V*_*cell*_ is the volume of the calorimetric cell and *ΔH*_*r*_ is the enthalpy change for the reaction. *ΔH*_*r*_ can be obtained integrating the signal from the calorimeter as:

$${\Delta H}_{r}\;=\;\frac{1}{n_{subs}}\int_{t=0}^{t=\infty }\frac{dQ}{dt}\;dt$$

with *n*_*subs*_ being the total number of moles of the substrate converted to product. The substrate concentration at any time during the course of the reaction is calculated from [13]:

$${\left[S\right]}_{t}\;={\left[S\right]}_{t=0}-\;\frac{1}{V_{cell}\;{\Delta H}_{r}}\int_{t=0}^{t}\frac{dQ}{dt}\;dt$$

In the ITC experiments, product inhibition can be assessed by performing a second injection after the reaction, occurring in first one has reached completion (return of the base line to the original power value). If the calorimetric trace is not identical in both injections, there is product inhibition.

### Analysis of product profile by HPAEC-PAD, HPLC and GPC

Monosaccharides, fructooligosaccharides and levan of low molecular weight were identified and analyzed by High Performance Anion Exchange Chromatography (HPAEC) with Pulsed Amperometric Detection (PAD) in a Dionex system (Thermo Scientific, USA) using a CarboPac^®^ PA-200 column (2 mm × 250 mm; Dionex) kept at 30°C with a continuous gradient of sodium acetate and NaOH mixture at a 0.5 mL/min flow rate: elution was applied starting with 5 mM sodium acetate and 150 mM NaOH increasing sodium acetate molarity to 100 mM in 25 min and to 400 mM in 85 min, afterwards returning to the initial conditions.

In order to identify some of the products the following standards were also analyzed: β2–1 fructooligosaccharides (OligoTech^®^; Elicityl, France) with degree polymerization (DP) of 3 to 10 and solutions of glucose (DP 1), fructose (DP 1), sucrose (DP 2), 1-kestose (DP 3), 6-kestose (DP 3), neokestose (DP 3), nystose (DP 4) and f-nystose (DP 5). Commercial Inulobiose was identified from Orafti P95 inulooligosaccharides and levanobiose as described by Porras-Domínguez et al. [14]

Levan molecular weight distribution was analyzed by Gel Permeation Chromatography (GPC) using an Ultimate 3000 RS System (Dionex, ThermoScientific, USA) equipped with a set of Ultrahydrogel Linear 500 columns (7.8 mm × 300 mm), using 100 mM NaNO_3_ as mobile phase with an isocratic 0.8 mL/min flow rate at 35°C.

Glucose, fructose and sucrose were identified and quantified by High Performance Liquid Chromatography (HPLC) in a Waters 600E System (Waters Corporation, MA, USA) with refractive index detector (Waters 410) equipped with a Prevail Carbohydrate ES columns (250 mm × 4.6 mm), applying acetonitrile:water (65:35, v/v) as mobile phase with an isocratic flow of 1 mL/min at 30°C.

## Results and Discussion

As already reported, SacB has an exo-type levanase activity [6,7]. However the properties of this activity have not been examined in detail and, to our knowledge, the evolution of the hydrolysis products never explored beyond initial rate experiments. Furthermore, the structural integrity of levan after its synthesis has not been considered, nor the role of this activity in the final product profile. In preliminary experiments and under the common SacB reaction conditions (1 U/ mL in the working buffer at 25°C), we found that while SacB releases 1 μmol of fructose per min from 100 mg/mL of sucrose, the enzyme releases 1/18 μmol of fructose from 100 mg/mL of LMw levan. This is a minor activity when during the first stage of reactions with sucrose as substrate, but it may become important when the enzyme finds similar concentration of both, sucrose and levan, towards the end of the reaction.

### Kinetic behavior of SacB levanase activity

**Fig 1:**
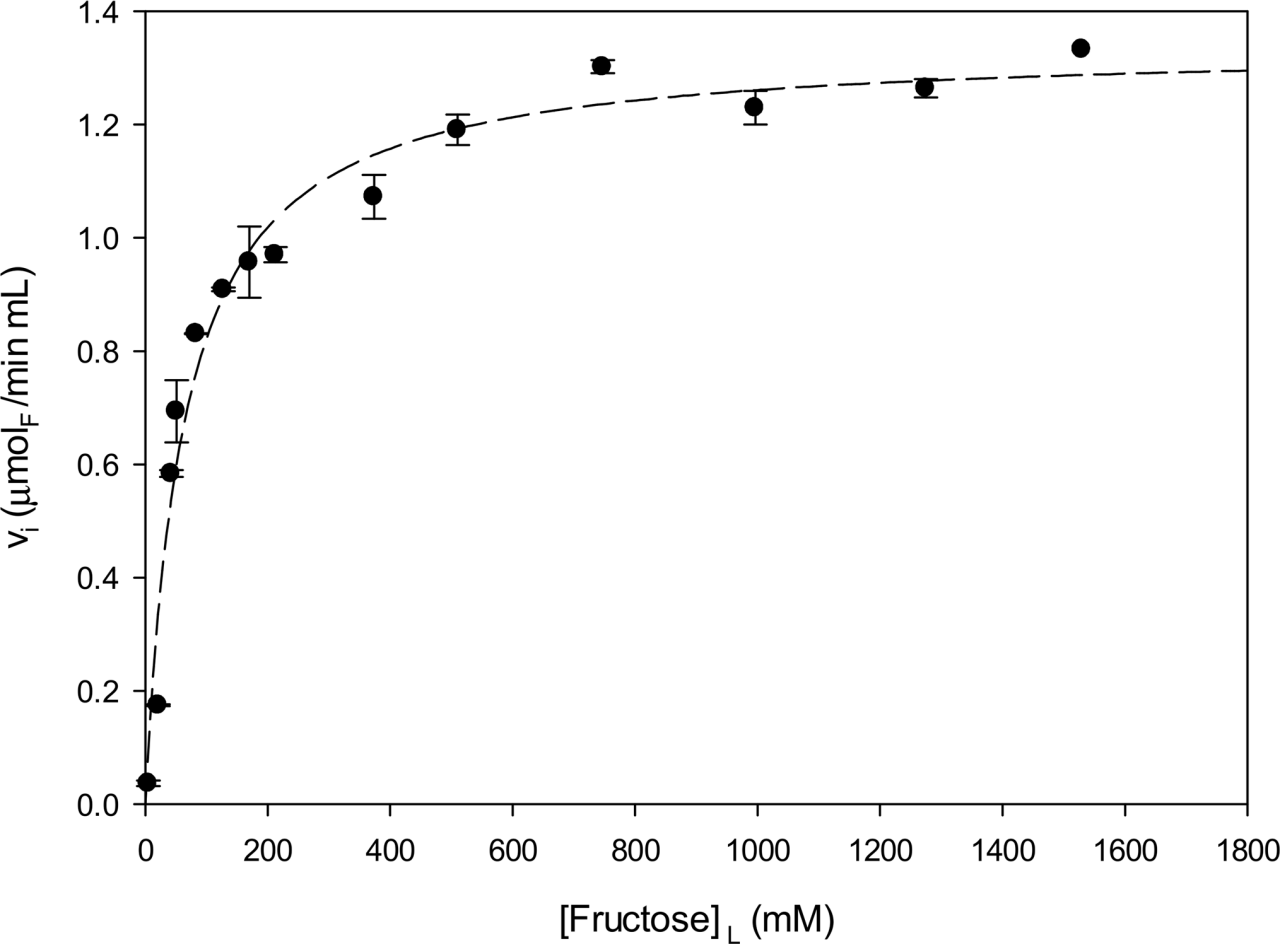
Initial levanase reaction rate of *B*. *subtilis* levansucrase as a function of low molecular weight levan concentration. The dash line represents the non-linear regression of the data using the Michaelis-Menten model. All reactions were carried out with 2.4 μM of SacB, at 37°C in the working buffer. Levan concentration is reported according to its total fructose concentration considering and average molecular weight of 8.3 kDa.

The levansucrase SacB showed optimal levanase activity in the pH range of 5 to 6, not surprisingly similar to the pH activity range reported for levansucrase activity with sucrose [4, 15]. Maximum levanase activity towards HMw and LMW levans was found in the range of 20 to 40°C. Initial reaction rates at different HMw and LMw levan concentrations were first measured in order to observe the kinetic behavior and determine the kinetic parameters that describe the hydrolytic activity of SacB. It was found that SacB follows Michaelis-Menten type kinetics when levan of low molecular weight is used as substrate as shown in Fig 1. By non-linear regression analysis of the data, a Michaelis Menten affinity constant of *K*_*m*_ = 63.4 mM, measured as fructose equivalents in levan, and a first order catalytic constant of k_*cat*_ = 9.3 s^-1^ were determined, with a correlation coefficient r^2^ = 0.97 (Fig 1). These results are differ from those values reported by Rapoport and Dedonder (1963) who found an affinity constant of 5 mM for levan of 8 kDa, by Yamamoto *et al*. (1985) who reported a catalytic constant of 5.7 x 10^−3^ s^-1^ at 30°C for levan of 10.9 ~ 34 kDa and from those of Chambert *et al*. (1974) who found an apparent limiting rate constant of 47 s^-1^ at 22°C for levans of 15 ± 3 kDa. In these reports, levanase activity was measured either by initial rate of reducing sugars release, or by the reduction of levan through turbidimetry.

As shown below, levan consists of a wide distribution of molecules, traditionally characterized by their GPC average Mw. Therefore these kinetic parameters are indicative only of the enzyme properties acting on the initial distribution of levan molecules used as substrate. In spite of this consideration, an interesting comparison results from SacB kinetic data on sucrose and levan [14, 15]: in terms of k_*cat*_ values, SacB is 17.7 times faster on sucrose (global levansucrase activity, k_*cat*_ = 164.6 s^-1^) than on levan (levanase activity, k_*cat*_ = 9.3 s^-1^), with an affinity 7.9 times higher for sucrose than for levan (63.4 mM/8 mM). In the same context, SacB hydrolyses sucrose 7.65 times faster than levan (71.2 s^-1^/ 9.3 s^-1^), with higher affinity for sucrose (*K*_*m*_ = 8 mM) than for levan (*K*_*m*_ = 63.4 mM).

**Fig 2:**
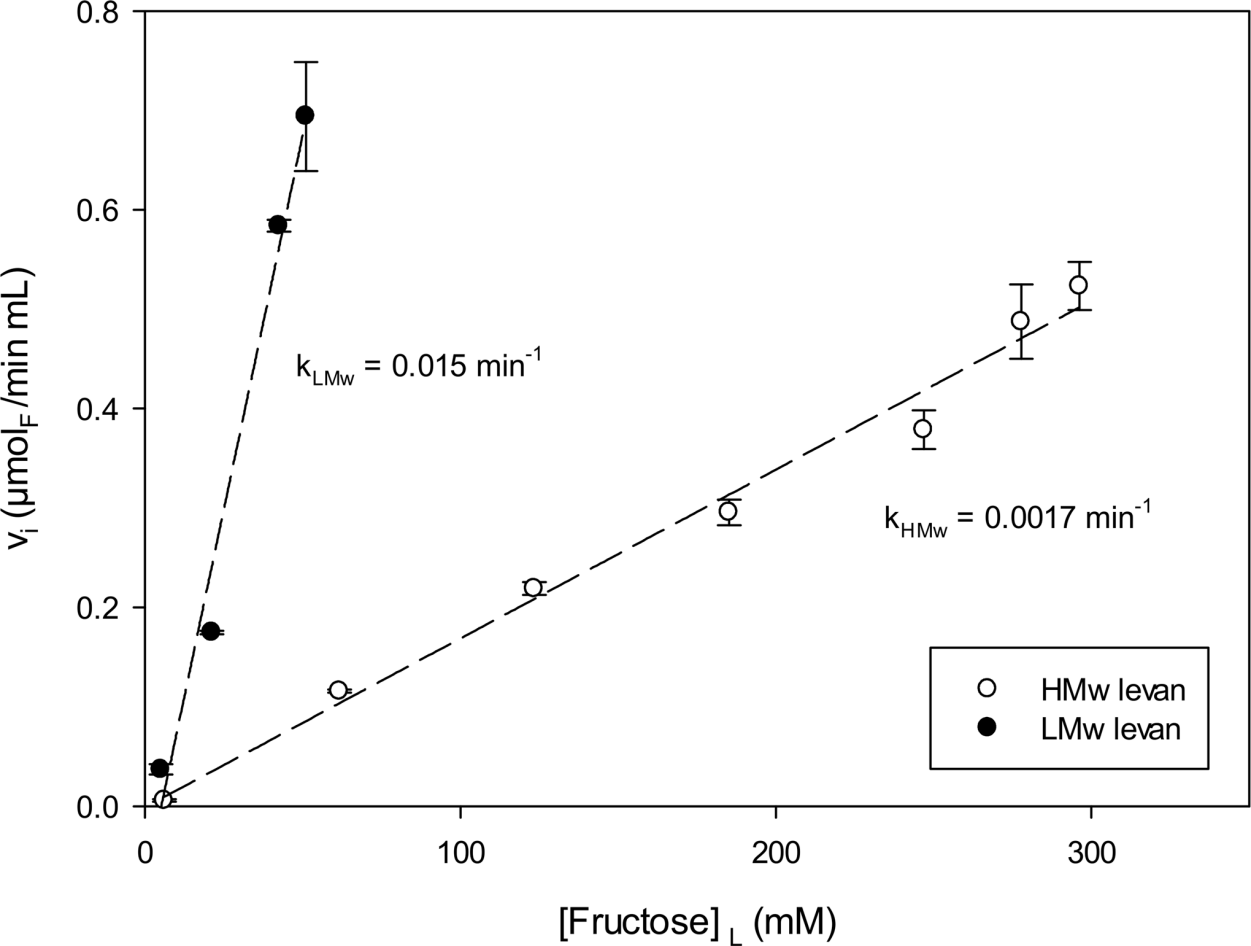
Kinetic behavior of SacB levanase activity towards high (HMw) and low (LMw) molecular weight levans, measured as initial rates at 37°C with 2.4 μM of enzyme in the working buffer. The concentrations were: 6 to 296 mM_F_ for HMw levan and 5 to 51 mM_F_ for LMw levan.

Fig 2 shows the initial SacB levanase activity with high molecular weight levan as substrate, here reported as the total fructose concentration in the reaction. In this case, a linear first order kinetic behavior is observed up to a total fructose concentration of 48 mg/mL; this is equivalent to an HMw levan concentration of 0.02 mM, the highest concentration of HMw levan than can be prepared. Nevertheless, if the rate of hydrolysis of both HMw and LMw polymers are compared at the same enzyme concentration (2.4 μM) in the first order region, the first order kinetic constant is 8.8 times higher for levan of low molecular weight, as shown in Fig 2. This is not unexpected, considering not only the differences in physicochemical properties between both levans (diffusivity, viscosity, solubility) but also the structure reported for HMw levan, with a large amount of branching [6, 11].

### Levan hydrolysis kinetics by ITC

Considering the already described kinetic evolution of the levanase reaction, which involves a continuous modification of the substrate (levan), it is evident that a better description of SacB kinetic behavior can only be obtained following the reaction rate evolution during the reaction time. Therefore, the reaction was studied in an Isothermal Titration Calorimeter, an equipment that allows the continuous measurement of the heat of reaction, which is proportional to the instantaneous reaction rate which, in turn, is a consequence -among other factors- of the levan molecular weight reduction and the increase in total substrate molar concentration. An additional factor to consider in the ITC analysis is the effect on reaction rate of the accumulation of reaction products.

From the calorimetric data of several ITC experiments, using equations (1) to (3) and assuming a simple Michaelis-Menten behavior the average steady state kinetic parameters k_*cat*_ and *K*_*m*_ for the hydrolysis of the low molecular weight levan were evaluated. An average *K*_*m*_ value of 1.325 mM, was found, a lower value that the *K*_*m*_ value determined from initial rate data (levan concentration measured as fructose equivalents). On the other hand, and as expected, the k_*cat*_ value is considerably lower (0.32 s^-1^) than the value obtained from initial rate data (9.3 s^-1^), as it includes not only the effect of product inhibition (see below), but also the fact that the initial levan distribution is modified as the reaction proceeds.

**Fig 3:**
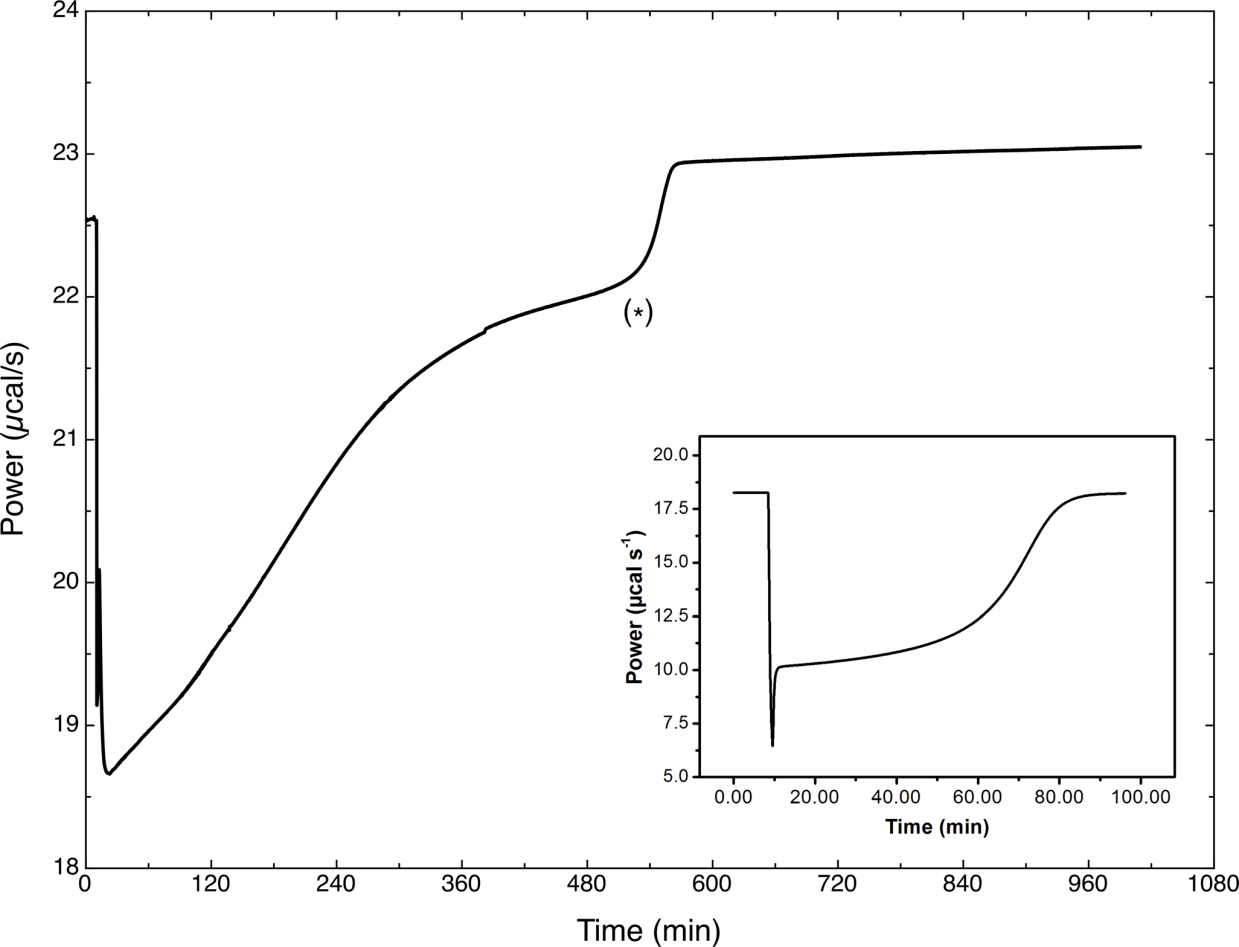
Calorimetric trace of LMw levan hydrolysis by *B*. *subtilis* levansucrase (SacB) in a VP-ITC. The enzymatic reaction was carried out at 25°C. The reaction took place in the 1.4 mL calorimetric cell, containing 500 nM SacB in the working buffer. The reaction started by the addition of 40 μL of a 30.16 mM LMw levan solution placed in the calorimeter syringe. After this titration the LMw levan concentration in the cell was 0.85 mM. (*) indicates the onset of the “second reaction” For comparison, the inset show a calorimetric trace for a single reaction to completion (for the hydrolysis of L-arginine ethyl ester catalyzed by tripsin from Martínez (2015)).

Fig 3 shows a typical raw data curve from the ITC. It is observed that the thermal power produced by the hydrolysis of the polysaccharide is large and so is the time required to reach a plateau indicating the end of the reaction (around 12 hours). A surprising phenomenon is observed 9 hours after the injection, as a sudden change in the heat profile takes place. This is a second step in the reaction taking place in the calorimetric cell, most probably resulting from the hydrolysis or disproportionation of the accumulated products as observed in the figure. The second stage in the heat profile of the hydrolysis reaction rapidly reaches a plateau, which is close to the original baseline, indicating reaction completion. A second injection (not shown) of substrate at this point produced a different calorimetric trace (different area and evolution with time) clearly indicating there is inhibition by products. From the ITC results, it is clear that there is only a partial hydrolysis of the substrate. The confirmation of the “second reaction” as well as an effort to identify its products is described below.

### Fructose release during levan hydrolysis

**Fig 4:**
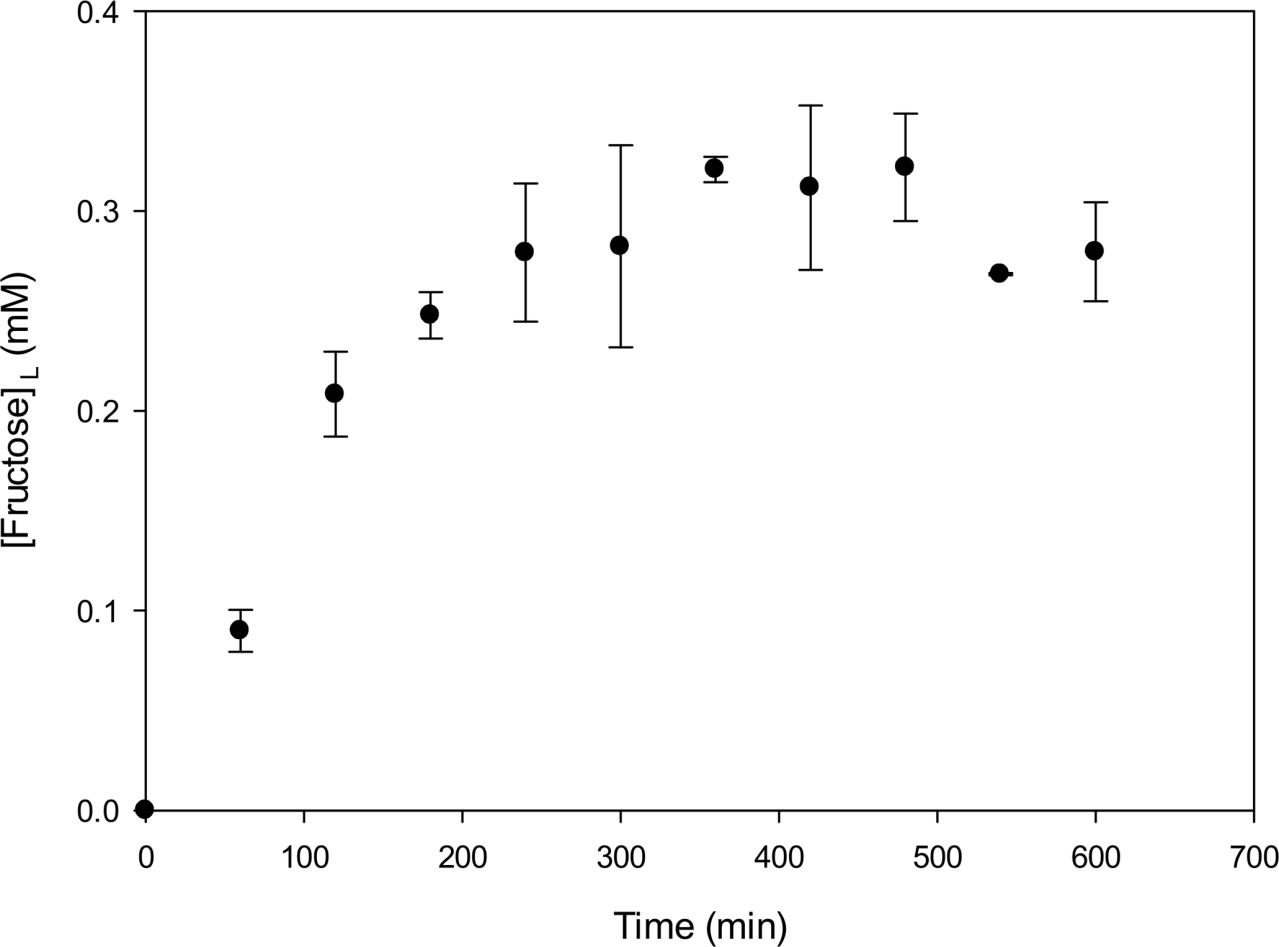
Evolution of fructose liberation from LMw levan hydrolysis by SacB (500 nM) at 25°C, with LMw levan (0.85 mM) in a total reaction volume of 1.4 mL in the working buffer. These are the same concentrations employed in the ITC experiment depicted in Fig 3.

The reaction observed in Fig 3 using ITC was also monitored measuring the reaction products released during 600 min by HPLC. As already reported, the main reaction product is fructose, resulting from an exo-type mechanism of levan hydrolysis [6, 9, 10]. The evolution of fructose release during the reaction is shown in Fig 4, where several regions may be identified. In fact, there is a linear increase in fructose accumulation during the first 240 min of reaction, followed by a progressive rate reduction until no more fructose seems to be released from levan. No inhibition effect of fructose on SacB reaction rate was observed (results not shown). It is important to mention that large deviation in the fructose measurements were obtained once the reaction reaches an apparent end, as also reported in Fig 4. It was found that the reaction stops when 33% of the total fructose mass has been released from levan. This limitation had also been reported by Rapoport and Dedonder (1963) with an 8 kDa levan and must probably derives from the fact that levan “limited structures” have been reached.

### Evolution of levan hydrolysis products by GPC

**Fig 5:**
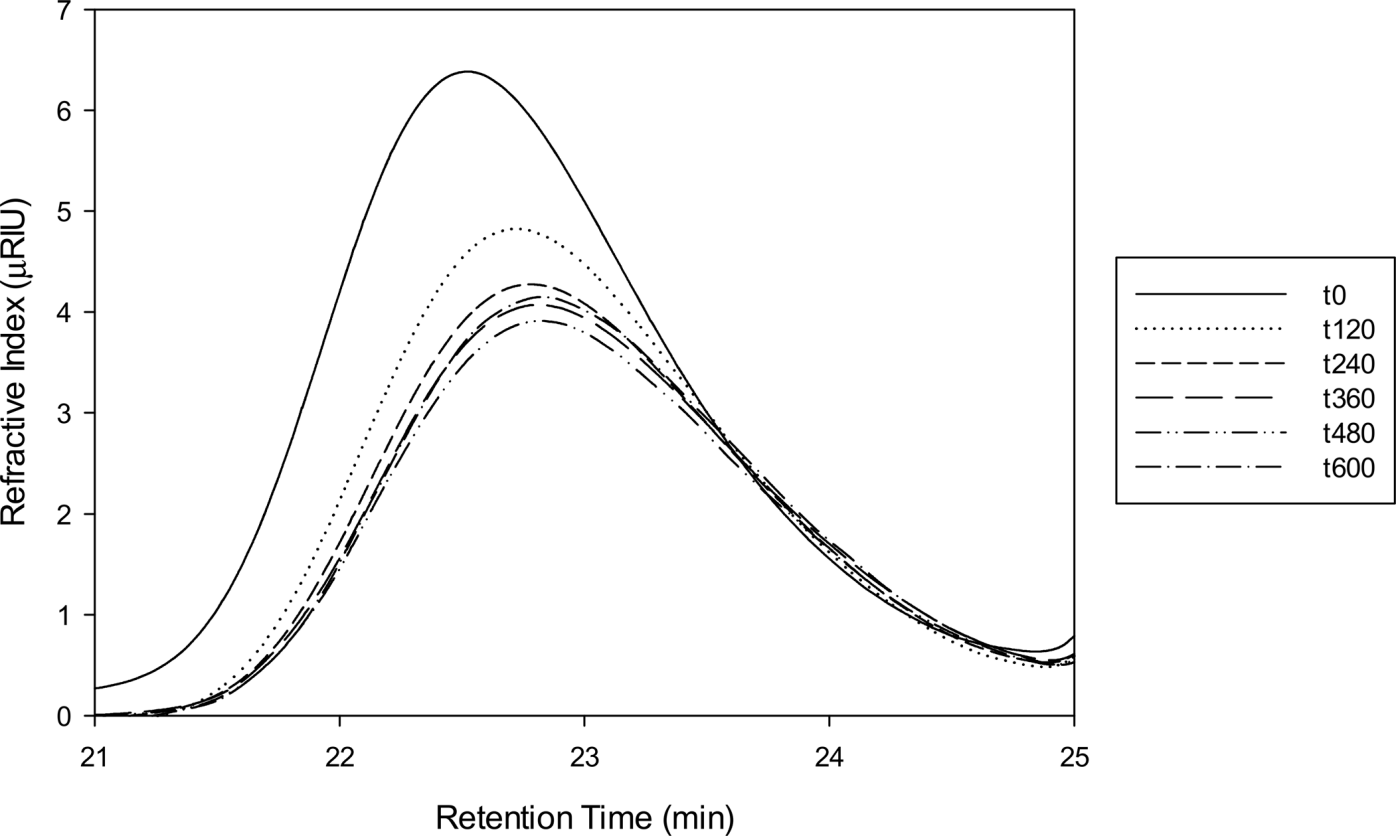
Evolution of low molecular weight levan during hydrolysis by SacB (500 nM) as determined by GPC. The reaction took place at 25°C with LMw levan (0.85 mM) in a total reaction volume of 1.4 mL in the working buffer. These are the same concentrations employed in the ITC experiment depicted in Figs 3 and 4.

Low molecular levan is constituted by a wide number of polymer molecules with a normal distribution of molecular weight with an average of 8.3 kDa as already described by [1]. Using GPC the modification of this distribution was observed during the hydrolysis reaction with SacB. In Fig 5, it may be observed that, as expected, there is a gradual reduction in the polymer molecular weight distribution, concomitant to the increase in fructose concentration reported in Fig 4. Nevertheless, although there is a clear reduction of low molecular weight levan molecules size and concentration, as deduced from the decrease in the area and elution time of the GPC distribution, there is a limit for this reduction, which match with the free fructose release during the reaction. As the addition of fresh enzyme does not restarts the hydrolysis, it seems logical to assume that a particular product structure no longer accessible to the enzyme has been reached, must probably related to branching points in the levan molecule. It has been reported that levan from different microorganisms contains around 2–30% branching β2–1 links [16–18]. We did not found SacB activity when the enzyme was essayed against linear β2–1 inulin (results not shown). However, once this limited hydrolysis state is reached, the addition of an exoinulinase for a short reaction time (10 min and later deactivated), modifies levan structure making it susceptible again to SacB, indicating the nature of the β2-1branching linkages that stops SacB exo levanase activity. At the end of this second SacB reaction a normal distribution of levan hydrolysis products is still observed.

### Evolution of levan hydrolysis products by HPAEC-PAD

**Fig 6:**
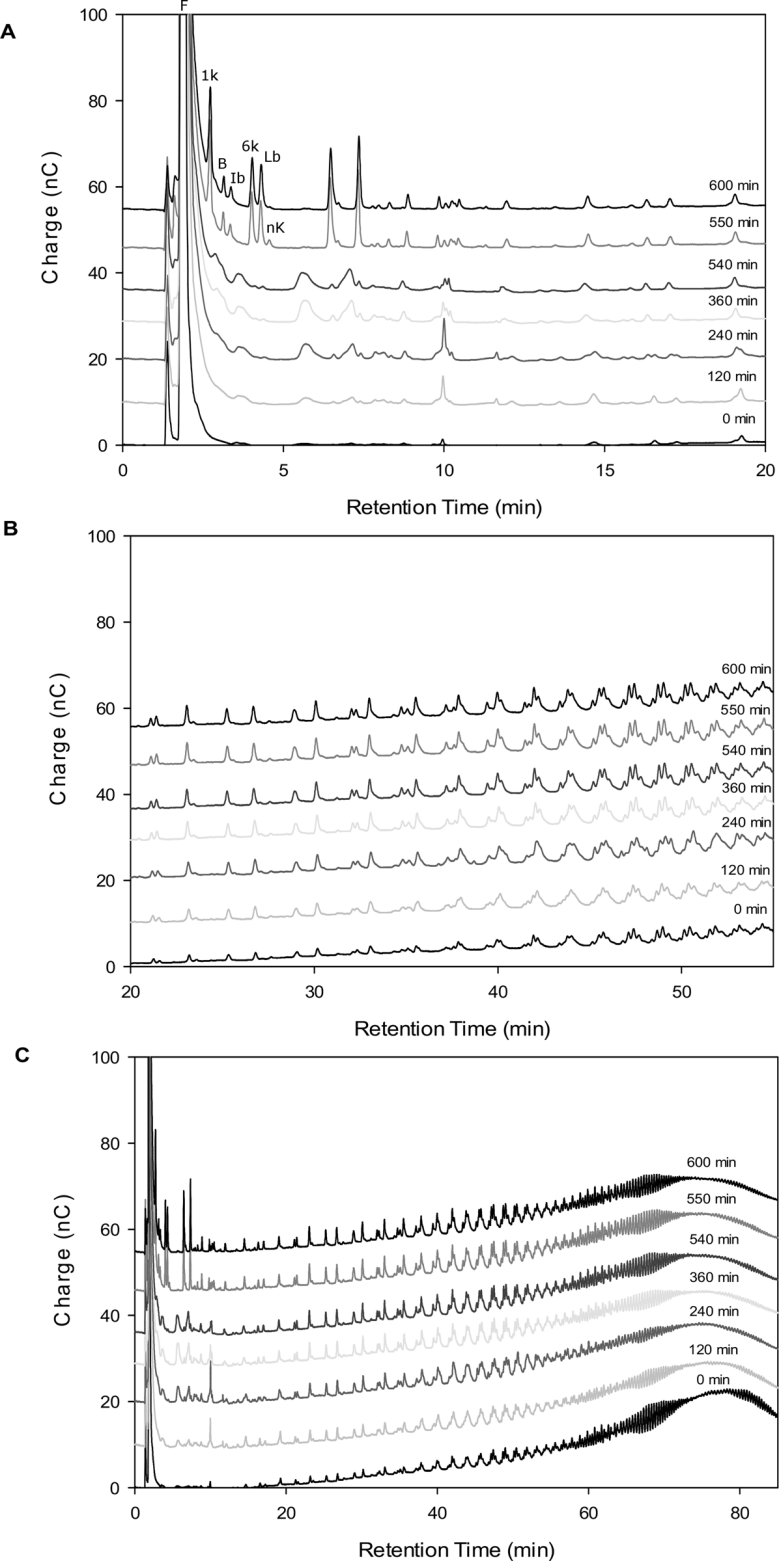
Evolution of low molecular weight levan distribution during hydrolysis by SacB (500 nM) as determined by HPAEC-PAD. Samples correspond to reactions also described by Figs 3, 4 and 5. The chromatograms are shown according to levans molecular weights:A) Mono-, di- and fructo-oligosaccharides with elution times of 1.6–20 min, B) intermediate size levans with elution times of 20–50 min, C) Chromatogram showing all reaction products obtained from levan. Fructose (F), blastose (B), 1-kestose (1k), 6-kestose (6k), levanobiose (Lb), inulobiose (Ib) and neokestose (nk).

A detailed characterization of the evolution of levan hydrolysis in the DP < 25 region, was carried out using HPAEC-PAD, during the reaction studied by calorimetry and GPC. The product profile evolution starting from LMw levan is described in Fig 6. As shown in this figure, LMw levan consists of a wide normal distribution of fructan molecules combining linear and branching structures of diverse molecular weight, with an average of 8.3 kDa. For the purpose of this analysis, the product distribution is divided in two sections: simple sugars and small fructooligosaccharides (DP 5 and lower) eluting during the first 15 min (Fig 6A) and medium size products with DP from 6 to 25 eluting between 16 and 55 min (Fig 6B). The overall reaction is depicted in Fig 6C.

In Fig 6A it may be observed that the smallest fructooligosaccharides (FOS) fraction increases and accumulates with reaction time up to 540 min. After 550 min of reaction, new products of low molecular weight are generated. Besides fructose, a single product with retention time of 10 minutes accumulates and decreases after 360 min of reaction. The product profile reached after 300 min is maintained until 540 min of reaction. Nevertheless, once 550 min of reaction are reached, there is an important modification in FOS profile as may be observed in Fig 6A in accordance to the calorimetric phenomenon already described. Products that had not changed for 240 minutes disappear, while 1-kestose (β-D-Fru*f*-(2→1)-β-D-Fru*f*-(2→1)-α-D-Glc*p*), blastose (β2,6 Fru-Glc), inulobiose (β2,1 Fru-Fru), 6-kestose (β-D-Fru*f*-(2→6)-β-D-Fru*f*-(2→1)-α-D-Glc*p*) and levanobiose (β2,6 Fru-Fru), followed by two unidentified products with elution times between (6 and 8 min) appear. These modifications are clear in the low DP fraction (Fig 6A). The last FOS profile is retained until 600 min of reaction. The second fraction of products, with intermediate size (6 < DP < 25) are only slightly modified, with minor changes in the profile (Fig 6B). Finally, the area of the peaks corresponding to larger DP levan molecules decrease with reaction time until around 240 min remaining constant afterwards, as already shown by GPC (Fig 5).

In general, HPAEC-PAD provide evidence of changes in product profile and their evolution in time, in agreement with the changes observed by calorimetry. As fructose concentration remains constant during the second reaction stage, the changes observed must correspond to a disproportionation reaction, in which the enzyme transfers a fructosyl residue from the non-reducing end of a donor levan chain to the non-reducing end of a different chain, to fructose or to an oligosaccharide. As already mentioned, this type of transferase activity was demonstrated in *B*.*subtilis* levansucrase by Rapoport and Dedonder (1963), but also for levansucrase from *L*. *reuteri* with inulin type FOS [9]. Similarly, amylosucrase from *Neisseira polysaccharea* synthesizes malto-oligosaccharides through disproportionation reactions [19]. The presence of these reactions may explain the high variability of free fructose concentration observed at the end of the first reaction stage (240min in Fig 4). However, this activity has not been studied directly using levan as donor and fructose as the acceptor molecule, as happens as the reaction from sucrose approaches the end. In this context, glucose could also be involved as an acceptor molecule.

### Glucose and fructose as acceptors of fructosyl transfer from levan

**Fig 7:**
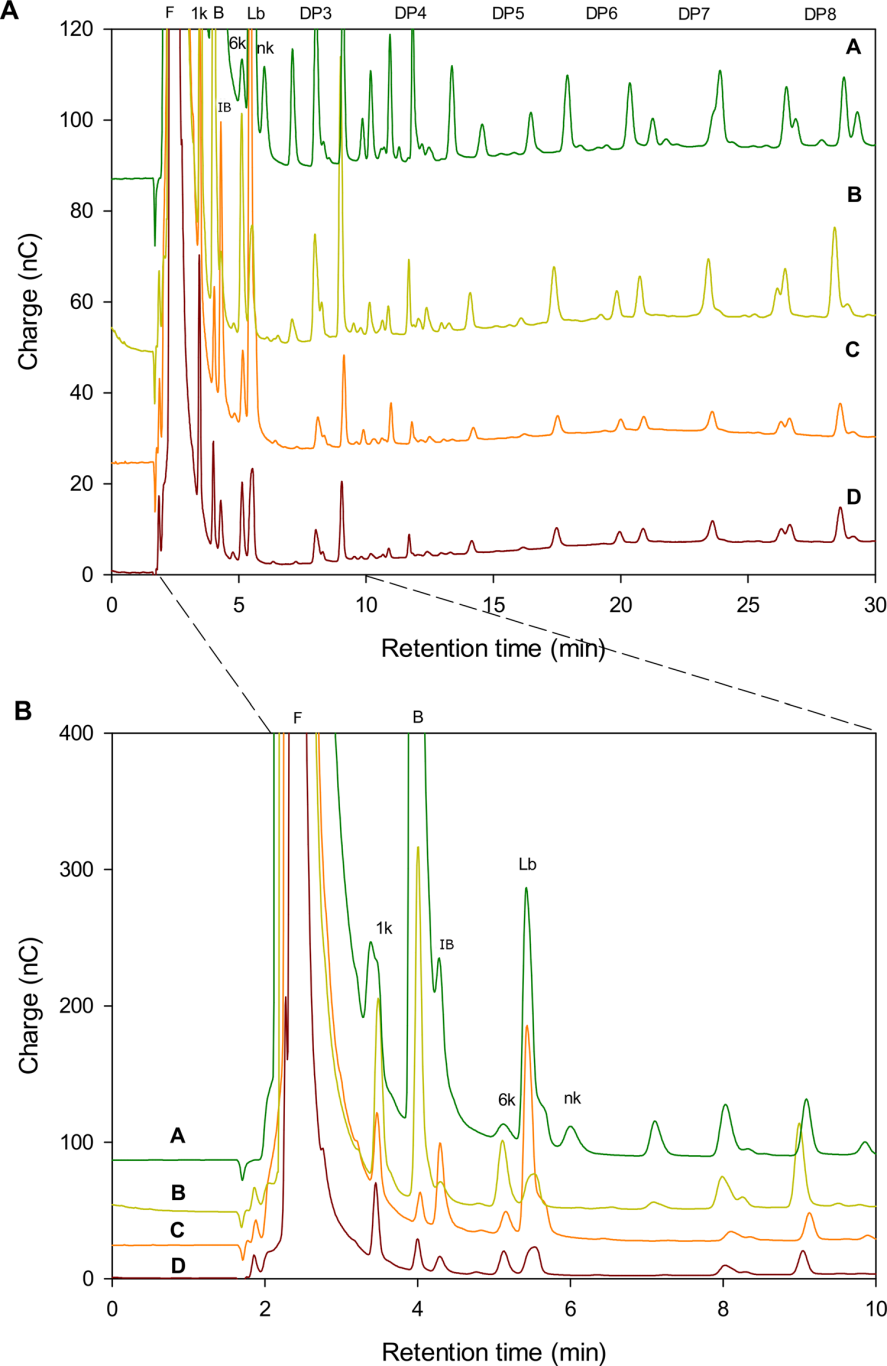
*B*.*subtilis* Levansucrase reaction products profile as observed by HPAEC-PAD: A) Products obtained from sucrose B) Products obtained from levan as donor and glucose as acceptor, C) Products obtained from levan as donor and fructose as acceptor, D) Levan hydrolysis products. Fructose (F), blastose (B), 1-kestose (1k), 6-kestose (6k), levanobiose (Lb), inulobiose (Ib) and neokestose (nk) were identified from standards. The DPn region is shown according to inulo-oligosaccharide standards.

To study the above-mentioned transferase activity, a reaction with glucose or fructose as acceptor was carried out in the presence of LMw levan as donor as described in M&M. After 5h of reaction, the samples were analyzed by HPAEC-PAD and compared to the fructooligosaccharides produced in the levan synthesis from sucrose and to the SacB levan hydrolysis profile (Fig 7). The product profile of a SacB reaction with levan in the presence of fructose (Fig 7, line C) shows a wide diversity of products, among which levanobiose is the most abundant, but also includes inulobiose (β2–1 Fru-Fru) and probably an F3 oligosaccharide (the peak eluted after 9min). However, a surprising result was obtained when glucose was used as acceptor. In this case the sucrose analogue blastose, as well as the trisaccharides 1-kestose and 6-kestose were identified as the most abundant products (Fig 7, line B). These profile results from the fructosyl transfer from levan to glucose either in β2–6 (blastose) or β2–1 to synthesize sucrose. Recently, Santos-Moriano et al. [20] proposed a similar acceptor reactions mechanism for levansucrase from *Zymmomonas mobilis* in which blastose and levanobiose are synthesized from glucose and fructose respectively, but no inulobiose was reported. The synthesized sucrose, as the highest affinity substrate is used as donor for the production of fructooligosaccharides similar to those observed when reactions are performed with sucrose as the sole substrate (Fig 7, line A). The polymerization degree of these products as estimated from the available DPn standards shows a diversity of fructooligosaccharides for each polymerization degree, as shown in the upper part of Fig 7. These results suggest a role of glucose and fructose in levan synthesis, through reactions in which the monosaccharides are used as acceptors. Although acceptor reactions with the monosaccharides were previously reported by Tanaka using sucrose as donor, only the reverse reaction was reported in the case of glucose and the synthesis of levanobiose and levanotriose from fructose [21], but no blastose nor inulobiose were reported.

In conclusion, SacB has an intrinsic levanase activity which was evaluated directly in the presence of levan. In this context, levan is hydrolyzed through an exo-type mechanism which is limited to a conversion extent of around 30% when all polymer molecules reach a structure no longer suitable to SacB hydrolysis, probably containing branches in β2–1 position. Alternatively, SacB reactions with levan in the presence of glucose and fructose, (a condition found during levan synthesis), results in the production of a wide variety of oligosaccharides such as blastose, levanobiose, inulobiose, 1 kestose and 6 kestose. As the transfer to glucose results in the synthesis of blastose and sucrose, a typical SacB reaction from its original substrate may also take place.
